# Persistent Vagal Activation Caused by a Large Mesenteric Hematoma After Blunt Abdominal Trauma: A Case Report

**DOI:** 10.7759/cureus.107608

**Published:** 2026-04-23

**Authors:** Kazuyuki Miyamoto, Hiroki Yamaga, Mako Sakakibara, Masaharu Yagi, Kenji Dohi

**Affiliations:** 1 Emergency, Critical Care Medicine and Disaster Medicine, Showa Medical University Fujigaoka Hospital, Yokohama, JPN; 2 Emergency, Critical Care and Disaster Medicine, Showa Medical University School of Medicine, Tokyo, JPN; 3 Emergency, Critical Care Medicine and Disaster Medicine, Showa Medical University, Tokyo, JPN

**Keywords:** blunt abdominal trauma, bradycardia, mesenteric hematoma, shock, vasovagal reaction

## Abstract

In patients with trauma, decreased circulating blood volume and pain increase sympathetic nerve activation, which increases the heart rate (HR) and vasoconstriction. When this compensatory mechanism is disrupted, the effects of trauma are more severe. Stimulation via the afferent vagal nerves from the gastrointestinal tract causes vagal activation, inducing a cardioinhibitory response that sometimes causes shock and bradycardia. A 51-year-old man sustained an abdominal injury while playing baseball. His blood pressure was normal; however, examination revealed persistent sinus bradycardia (HR: 38-42 bpm). Focused assessment with sonography for trauma (FAST) examination revealed fluid accumulation in the hepatorenal space, suggesting intraperitoneal hemorrhage, which was subsequently confirmed by contrast-enhanced CT. After arrival at the hospital, the patient’s HR suddenly decreased to 32 bpm, and his blood pressure dropped to 58/40 mmHg, consistent with shock. He was diagnosed with a vasovagal reaction and recovered after receiving two doses of atropine. Contrast-enhanced CT revealed a large mesenteric hematoma without extravasation of contrast media. After the CT examination, the patient’s HR again suddenly decreased to 36 bpm, and his blood pressure dropped to 83/42 mmHg with peripheral coldness, indicating recurrent shock. After the administration of atropine and continuous dopamine, his vital signs stabilized. A laparotomy revealed that the mesentery of the descending colon was filled with a large hematoma (820 g), with an estimated blood loss of 1100 mL. The descending colon was partially resected. The bradycardia did not recur after surgery. Vagal afferent fibers terminate in the mesentery and monitor tension. In our patient, the mesentery was stretched by a large hematoma, which induced persistent vagal activation and sinus bradycardia. Owing to vagal activation, the compensatory mechanism was not fully functional. Repeated vasovagal reactions combined with blood volume loss can cause shock. Therefore, when a patient presents with persistent bradycardia following trauma, the possibility of a vasovagal reaction should be considered.

## Introduction

In patients with trauma, decreased circulating blood volume and pain increase sympathetic nerve activation, resulting in an increased heart rate (HR), vasoconstriction, and increased ventricular contractility [1]. This compensatory physiological response is life-preserving in cases of injury. Hemorrhage accounts for 30-40% of trauma-related deaths and represents the second most common cause of early traumatic mortality after central nervous system injury [2]. Tachycardia is typically the earliest and most reliable sign of hemorrhagic shock, and its absence is often interpreted as evidence of hemodynamic stability. However, this assumption can be dangerously misleading in patients with concurrent vagal activation, where bradycardia may paradoxically coexist with significant blood loss and hemodynamic compromise.

Bowel and mesenteric injuries occur in approximately 5% of patients who sustain blunt abdominal trauma and are among the most frequently missed injuries in this setting [3]. Motor vehicle collisions are the most common mechanism, but sports-related trauma is also a recognized cause [4]. Delayed diagnosis of mesenteric injuries is associated with significantly increased morbidity, including prolonged hospital and intensive care unit stays [5].

Physiologically, stimulation of afferent vagal nerve fibers from the heart, great vessels, eyes, and gastrointestinal tract causes vagal activation. It induces a cardioinhibitory response that can lead to sinus bradycardia, PR interval prolongation, advanced atrioventricular block, or asystole [6], and sometimes causes shock due to the vasovagal reaction. Vasovagal reactions may result in severe shock in patients with trauma because they disrupt the compensatory physiological responses to injury [7]. Therefore, recognizing vagal activation at an early stage is important in patients with trauma. In the emergency setting, trauma-related shock is typically associated with tachycardia as part of the compensatory sympathetic response. However, some patients paradoxically develop marked bradycardia and hypotension due to vagally mediated reflexes, which can be easily mistaken for hemorrhagic shock and may lead to delayed or inappropriate management. This case report highlights the diagnostic challenge posed by persistent bradycardia in a trauma patient--a presentation that deviates fundamentally from the classic paradigm of hemorrhagic shock--and underscores the importance of considering vagally mediated mechanisms when hemodynamic instability appears disproportionate to the estimated blood loss.

## Case presentation

**Figure 1:**
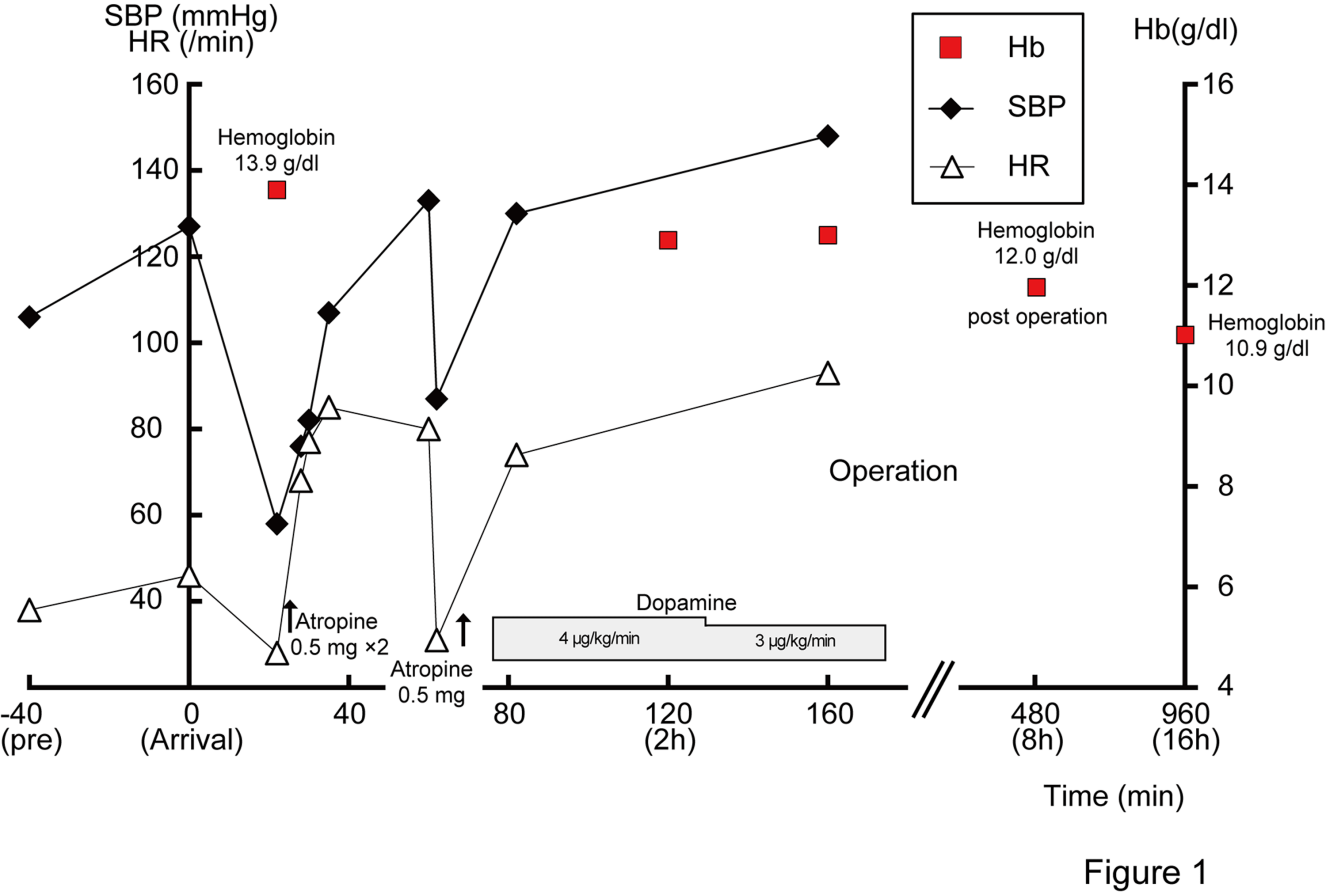
Time course of systolic blood pressure (SBP), heart rate (HR), and hemoglobin (Hb) in the patient Note the persistent bradycardia from prehospital presentation through admission, the transient hemodynamic recovery following atropine administration, and the recurrent shock after CT examination. The absence of a compensatory tachycardic response throughout the clinical course is consistent with sustained vagal activation. Hemodynamic stabilization was achieved only after surgical decompression of the mesenteric hematoma.

A 51-year-old healthy man (height: 172 cm, weight: 71.2 kg, BMI: 24 kg/m2) collided with a teammate during a baseball game and was struck on his abdomen. He felt intermittent abdominal pain and nausea an hour later. Ambulance crew reported his blood pressure (BP) (116/63 mmHg) was normal; however, sinus bradycardia (42 beats/min) existed (Figure 1).

On arrival at our emergency department, the patient was triaged as urgent (category II) owing to persistent bradycardia despite a normal blood pressure. After arriving at the hospital, his BP (127/89 mmHg) was unchanged, and sinus bradycardia (51 beats/min) persisted. The patient reported dull pain in his left lower abdomen. There was tenderness present, although rebound tenderness or abdominal muscular defense to indicate peritonitis was not found. We administered acetaminophen (1000 mg) to relieve the symptoms. Focused assessment with sonography for trauma (FAST) showed fluid accumulation in the hepatorenal space; therefore, intraperitoneal hemorrhage due to blunt abdominal trauma was suspected, which was subsequently confirmed by contrast-enhanced CT. The patient had no history of medication use, including beta-blockers or antiarrhythmic agents. Electrocardiography demonstrated sinus bradycardia without conduction abnormalities, and laboratory tests revealed no electrolyte abnormalities or other identifiable causes of bradycardia. Blood count showed that the hemoglobin (Hb) level was 13.9 g/dl, and there was no abnormality in other laboratory data.

**Figure 2:**
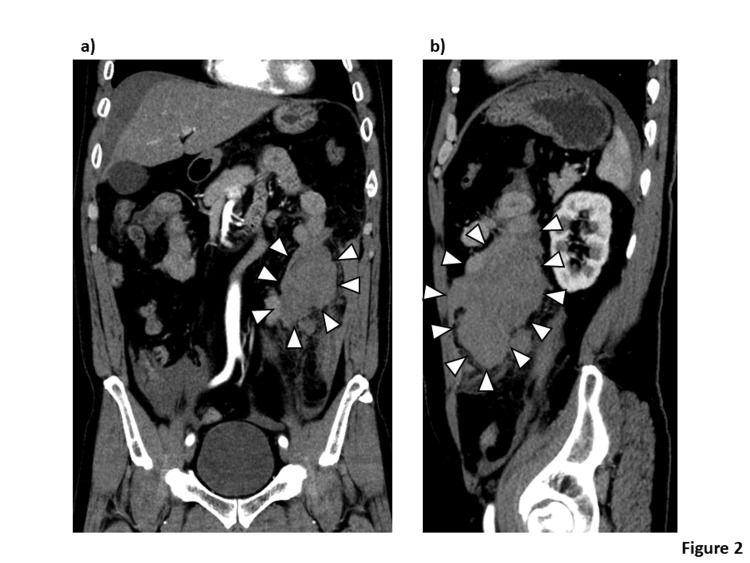
Contrast-enhanced computed tomography from the chest to pelvis: (a) coronal section, (b) sagittal section A large mesenteric hematoma (arrowhead) and intra-abdominal hemorrhage are present, without extravasation of the contrast media. The absence of active contrast extravasation indicates no ongoing arterial hemorrhage, supporting the conclusion that the volume of blood loss alone was insufficient to account for the severity of hemodynamic compromise. The large mesenteric hematoma is the proposed source of sustained mesenteric stretching and vagal activation.

Before moving to computed tomography (CT), the patient suddenly complained of discomfort. The HR dropped to 32 beats/min on the monitor, and a junctional rhythm was found on the electrocardiogram. His BP dropped to 58/40 mmHg, consistent with shock, although cardiac wall motion was normal in the echocardiogram. Therefore, we diagnosed him with shock due to a vagal attack. We immediately performed rapid infusion and administered atropine (0.5 mg) twice, following which he gradually recovered from shock. Subsequent imaging with a contrast CT from chest to pelvis (Figure 2) revealed the large mesenteric hematoma and intra-abdominal hemorrhage, though there was no extravasation of contrast media.

**Figure 3:**
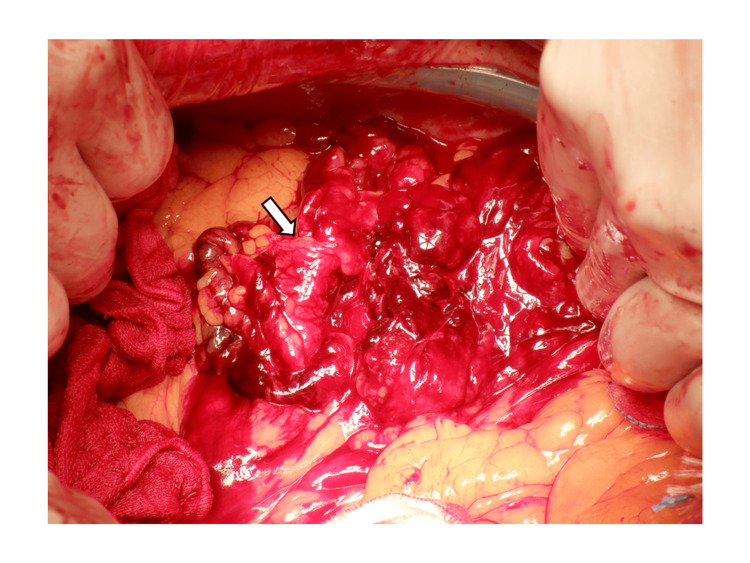
Intra-operative photographs of the abdominal cavity showing a large hematoma, weighing 820 g, in the mesentery of the descending colon The substantial size of the hematoma explains the degree of mesenteric distension and sustained vagal afferent activation. Complete surgical decompression resulted in immediate and lasting resolution of the bradycardia.

After returning from CT, the HR suddenly decreased again to 46 beats/min, and his blood pressure dropped to 87/56 mmHg with peripheral coldness, indicating recurrent shock. In addition to rapid infusion, we administered a continuous dose of dopamine (4 μg/kg/min), following which the patient’s HR and BP rose promptly and stabilized. Re-examined blood count showed that the Hb level was 12.9 g/dl; FAST showed no obvious increase in fluid accumulation. The degree of hemodynamic instability was considered disproportionate to the estimated blood loss, raising suspicion for a vagally mediated mechanism as a contributing factor. Given the persistent hemodynamic instability and large mesenteric hematoma on CT, a decision was made to proceed with emergency laparotomy. Intra-operative findings (Figure 3) showed that the mesentery of the descending colon was filled with a large hematoma, and the amount of blood hemorrhaged into the abdominal cavity was 820 ml. Partial resection of the descending colon was performed due to weak beating of the marginal artery. The mucosal surface of the descending colon was normal. Re-examined blood counts after surgery and 1 day post-surgery were 12 g/dl and 10.9 g/dl, respectively. The patient did not experience any further episodes of bradycardia during admission and was discharged home 15 days after surgery and resumed work (Figure 1).

## Discussion

Vagal afferent fibers generally terminate in multiple mechanosensitive endings in the mesentery, where they monitor tension on the mesenteric attachments [6]. Mechanical stimulation of the mesentery increases afferent vagal activation. Vasovagal reactions sometimes occur during colonoscopy or CT colonoscopy because of mesenteric stretching or colonic distension [8,9]. Sharma et al. reported sinus arrest that lasted 62 s and induced shock during colonoscopy [10], and Vayer et al. reported a vagally mediated bradycardic response following intraperitoneal injury [7]. In our patient, the mesentery was stretched by a large hematoma, inducing sustained vagal activation and persistent sinus bradycardia. Unlike vasovagal reactions during colonoscopy, which are transient and resolve upon cessation of the procedure, the mesenteric stretching caused by the hematoma produced a sustained vagal stimulus that could only be terminated by surgical decompression. A comparable case was reported by Rana et al., who described persistent paradoxical bradycardia in a patient with a complex splenic tear following blunt abdominal trauma [11]; however, in that case, the bradycardia was attributed to the volume of hemorrhage itself, whereas in our case, mesenteric stretching was the primary vagal trigger, as confirmed by the complete resolution of bradycardia after surgery.

The most common cause of shock in patients with trauma is hemorrhage. However, in this case, the estimated blood loss was approximately 1100 mL, calculated using the Gross formula (EBL = EBV × (Hb_initial − Hb_final) / Hb_initial) with an estimated blood volume of 5,477 mL (71.2 kg ÷ 13) [12]. Although this formula assumes normovolemia and does not fully account for hemodilution, the absence of active contrast extravasation on CT and the modest degree of anemia confirm that blood loss alone was insufficient to account for the hemodynamic compromise [1]. Persistent vagus nerve activation due to the mesenteric hematoma interfered with the normal compensatory mechanisms and led to shock despite the modest blood loss. Furthermore, unlike colonoscopy-related vasovagal reactions occurring in euvolemic patients, the blunted sympathetic response in our patient was superimposed on an already compromised compensatory reserve, amplifying the hemodynamic instability.

Atropine is the first-line treatment for vasovagal reactions, increasing HR and conduction velocity by preventing cholinergic activation. Santini et al. reported its efficacy in the cardio-inhibitory form of vasovagal reaction [13]. In our patient, atropine temporarily improved bradycardia and shock; however, recurrence occurred owing to its short-term effect of 0.7-4 min [13], necessitating continuous dopamine infusion as a bridge to surgery.

In the emergency setting, a key diagnostic dilemma is the co-existence of intraperitoneal hemorrhage and vagal activation, as both can cause hypotension and are difficult to distinguish clinically. A critical pitfall is that the absence of tachycardia may be falsely reassuring, potentially leading to delayed surgical intervention, and transient improvement following atropine may mask the persistence of the underlying vagal trigger. When vagally mediated shock is suspected, particularly when bradycardia persists or recurs and hemodynamic instability appears disproportionate to the estimated blood loss and imaging findings, continuous cardiac monitoring, prompt atropine administration, vasoactive support, and early surgical correction of the underlying trigger are essential.

## Conclusions

In this patient, a large mesenteric hematoma after blunt abdominal trauma caused persistent vagal activation, which led to decreased cardiac contraction and bradycardia. A combination of vasovagal reaction and a loss of blood volume can cause shock. Therefore, when a patient who has undergone trauma presents with bradycardia, the possibility of vagal activation should be considered. In particular, persistent or recurrent bradycardia in the setting of trauma, especially when the severity of hemodynamic instability appears discordant with the measured blood loss and imaging findings, should prompt clinicians to consider vagally mediated mechanisms alongside hemorrhagic shock, and to pursue early identification and surgical correction of the underlying trigger.
